# Comparative Evaluation of Urine, Sputum, and BALF for Human Microplastic Exposure Monitoring Using a Multicriteria Decision Framework

**DOI:** 10.1155/jt/5139238

**Published:** 2026-07-10

**Authors:** Neamatollah Jaafarzadeh Haghighi Fard, Faezeh Jahedi, Afshin Takdastan, Mehdi Ahmadi, Maryam Haddadzadeh Shoushtari

**Affiliations:** ^1^ Environmental Technologies Research Center, Medical Basic Sciences Research Institute, Ahvaz Jundishapur University of Medical Sciences, Ahvaz, Iran, ajums.ac.ir; ^2^ Student Research Committee, Ahvaz Jundishapur University of Medical Sciences, Ahvaz, Iran, ajums.ac.ir; ^3^ Department of Environmental Health Engineering, School of Health, Ahvaz Jundishapur University of Medical Sciences, Ahvaz, Iran, ajums.ac.ir; ^4^ Air Pollution and Respiratory Disease Research Center, Ahvaz Jundishapur University of Medical Sciences, Ahvaz, Iran, ajums.ac.ir

**Keywords:** AHP, BALF, biomonitoring, MCDA, microplastics, sputum, urine

## Abstract

Microplastic (MP) contamination in human biological systems has become a global health concern. However, the selection of the most suitable matrix for biomonitoring remains an open area of investigation. This study evaluates three biological matrices—urine, sputum, and bronchoalveolar lavage fluid (BALF)—for their suitability in MP detection and monitoring. Using data collated from a recent observational study (30 patients) and peer‐reviewed literature, we assessed detection sensitivity, polymer diversity, sample availability, and practical feasibility via a multicriteria decision analysis (MCDA) framework inspired by the analytic hierarchy process (AHP). Results showed that sputum exhibited the highest median MP concentration (9.4 particles/mL) and BALF showed the greatest polymer diversity (6 polymer types), while urine showed the lowest levels (2.7 particles/mL). The composite MCDA scores ranked urine highest (26/35) for population studies due to its noninvasiveness and reproducibility, followed by sputum (25/35) for respiratory exposure assessment and BALF (21/35) for deep‐lung mechanistic studies. We conclude that a matrix‐specific strategy is essential for advancing human MP biomonitoring.

## 1. Introduction

Microplastics (MPs) are called tiny plastic particles with sizes less than 5 mm [1, 2]. They have become worldwide contaminants of great persistence encountered in a variety of environmental matrices, ranging from marine and freshwater [3] to air, soil, and food systems [4–7]. Because of size, chemical stability, and adsorption abilities, MPs might easily interact with both environmental and biological systems [8].

MPs’ presence can be described in terms of inputs through inhalation of airborne MPs, ingestion of contaminated water and food, and skin absorption, the latter under some occupational or environmental conditions notwithstanding [9]. MPs have been detected ever since inside blood [10, 11], lungs [12, 13], placenta [14–16], stool [17], and semen [18, 19], indicating their dispersion to cover bigger areas and their ability to bioaccumulate.

This kind of evidence has in fact given rise to concerns about the toxicological effects of MPs, including chronic inflammation, oxidative stress, endocrine disruption, and genotoxicity [20–22]. In addition, the ability of MPs to carry pathogens and chemical additives particularly accentuates their health risk profile of concern in conjunction with vulnerable exposed populations [23, 24].

For assessing exposures to MPs, human body fluids, such as urine, sputum, stool, saliva, and bronchoalveolar lavage fluid (BALF) [13], among others, have been examined [25–27]. Each matrix offers its own merits and demerits. Urine contains MPs that have been filtered through the kidney, sputum shows upper respiratory clearance, and BALF is collected from the deep lung. However, existing literature is mostly geared toward detection rather than relative comparison of these matrices. The researchers included in our earlier survey study focused mainly on urine (Pironti et al. [28]; Rotchell et al. [27]). They reported low‐to‐moderate levels of MPs, mostly smaller than 100 µm in size. This size range may be relevant to renal filtration capacity, as particles of this dimension could potentially pass through the glomerular barrier. [27–29] [26, 30], mucus samples showed levels that had a stronger fibrous dominance—which can be attributed to airborne fibers that are incorporated into the air inhaled and which have been retained within the bounds of the upper airways. Studies utilizing BALF [31, 32] depict a range of MPs, showing levels of abundance that suggest previously encountered exposures from the deep lung, as well as a broader exposure to a range of environmental sources. Yet, no study has undertaken a comparison under the same methodological circumstances between these biological fluids, in order to evaluate which matrix best represents a biomarker for an exposure assessment of MPs. Our study documented MPs, abundance, and character in urine, sputum, and BALF of 30 patients with respiratory diseases. Although abundance and character were different across these matrices, their biomonitoring potential was not.

Therefore, in this study, we will revisit the published literature to (i) compare MP abundance, size, shape, and polymer characteristics in urine, sputum, and BALF; (ii) examine the pros and cons of matrices as biomonitoring; and (iii) give systematic suggestions for future human MP exposure studies.

To this end, we utilize a multicriteria approach, specifically analytic hierarchy process (AHP), to compare and rank the three matrices, in a systematic way, according to analytical performance and usability.

## 2. Materials and Methods

### 2.1. Study Design and Sample Source

This study is a secondary comparative analysis and methodological framework development. The primary data on MP abundance, size, shape, and polymer type in urine, sputum, and BALF were obtained from our previously published observational study [33]. That original study involved sample collection from 30 patients and received ethical approval from Ahvaz Jundishapur University of Medical Sciences (approval code: ETRC‐0302). The present study did not involve any new sample collection or human intervention; therefore, no additional ethical approval was required. The use of secondary data was intentional, as the original study did not perform a comparative multicriteria analysis of the three matrices for biomonitoring purposes. Thus, this study fills that gap by reanalyzing existing data through an AHP‐inspired decision framework.

### 2.2. Sample Collection and Preparation

Since our study is examining biological samples collected using standard clinical sample collection methods, strictly following the sample collection protocols of the previously mentioned study was not necessary. First, the urine samples were collected in sterile polyethylene (PE) tubes, and the sputum samples were collected by deep cough in sterile tubes. The BALF samples were collected by bronchoscopy in which saline was used to lavage the bronchi. All samples underwent enzymatic and chemical digestion (H_2_O_2_ and proteinase K) in order to lyse organic matter. Samples were then filtered on glass fiber membrane (1.2‐μm pore size), dried, and maintained under sterile conditions.

### 2.3. MP Identification

MP particles were distinguished through a stereomicroscope, and a subsample of particles was confirmed through FTIR. Particles were classified by shape (fiber, fragment, and bead), color, size (split by 10–50 μm, 51–100 μm, and > 100 μm), and polymer type.

#### 2.3.1. Quality Assurance and Contamination Control

To evaluate possible contamination, controls consisting of double‐distilled water (for urine), ethanol (for sputum), and isotonic saline (for BALF) were processed in parallel with all samples. Microscopic inspection of the control filters revealed 0, 12, and 3 fibrous particles per 10 mL for urine, sputum, and BALF controls, respectively. These values, and their distribution between the two size categories, were used to correct MP concentrations in the corresponding samples.

The limit of detection (LOD) was defined based on the control counts. Recovery efficiency was previously assessed in our laboratory by spiking clean filters with known quantities of PE and polystyrene (PS) microspheres (sizes: 10–50 μm), yielding a mean recovery of 87% ± 6% (*n* = 5). All reported concentrations were blank‐corrected.

### 2.4. Data Extraction and Statistical Analysis

Given that this study was a secondary analysis, we used the previously published data on MP characteristics (particles per mL, size distribution, and polymer type) from peer‐reviewed articles from each biological matrix. These data were organized and managed in SPSS (v26) for statistical comparisons.

The Shapiro–Wilk test was used to test normality of distribution. As data were not normally distributed, inter‐matrix comparisons were conducted with the Kruskal–Wallis H test, and this was followed by Dunn’s post hoc tests for pairwise analysis. A *p* < 0.05 was taken for statistical significance.

In addition to conventional statistical methods, a multicriteria decision analysis (MCDA) was also utilized based on a simplified scoring system inspired by the AHP. Seven criteria—detection sensitivity, diversity of polymers, ease of collection, noninvasive nature, biological significance, analytical interference, and repeatability—were qualitatively scored for each matrix on a 1–5 scale. Radar charts were employed to display scores to facilitate comparative interpretation and inform matrix‐specific recommendations.

## 3. Results

In this section, we present an analytical and interpretative comparison of the three biological matrices—urine, sputum, and BALF—for their suitability in biomonitoring MPs.

### 3.1. Relative Detection Efficiency

**FIGURE 1:**
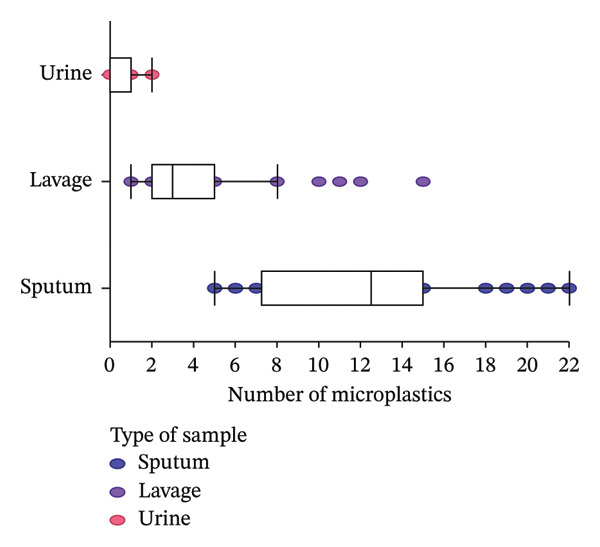
Distribution of MP concentrations (particles/mL) in urine, sputum, and BALF samples. Bars represent median values; error bars indicate interquartile range (IQR).

The highest median MP concentrations were observed in sputum (9.4 particles/mL), followed by BALF (6.2 particles/mL) and urine (2.7 particles/mL) (Figure 1). The Kruskal–Wallis analysis indicated significant differences between matrices (*p* < 0.05), with Dunn’s post hoc tests confirming that sputum was significantly different from urine (*p* = 0.002) and that urine was significantly different from BALF (*p* = 0.031). However, differences between BALF and sputum were not statistically significant (*p* = 0.072) (Table 1).

**TABLE 1:** Summary of descriptive and comparative statistics for MP concentration (particles/mL) in urine, sputum, and BALF.

Parameter	Urine	Sputum	BALF	*p* value (Kruskal–Wallis)
Median (particles/mL)	2.7	9.4	6.2	
Interquartile range (IQR)	2.1–3.5	8.0–10.7	5.1–7.5	
Mean ± SD	2.9 ± 1.1	9.6 ± 1.9	6.3 ± 1.6	< 0.05
Pairwise significance (Dunn’s post hoc)				
Urine vs. sputum	—	Significant (*p* = 0.002)	—	
Urine vs. BALF	—	—	Significant (*p* = 0.031)	
BALF vs. sputum	—	Not significant (*p* = 0.072)	—	

*Note:* Data extracted from [33]. Pairwise comparisons were performed using Dunn’s post hoc test following Kruskal–Wallis H test.

Abbreviations: IQR, interquartile range; SD, standard deviation.

This suggests that sputum provides higher sensitivity for MP detection, especially for particles retained in the upper respiratory tract. Its advantage lies in being a noninvasive yet lung‐proximal fluid, making it a strong candidate for short‐term inhalation exposure assessment.

### 3.2. Particle Characteristics and Interpretative Trends

**FIGURE 2:**
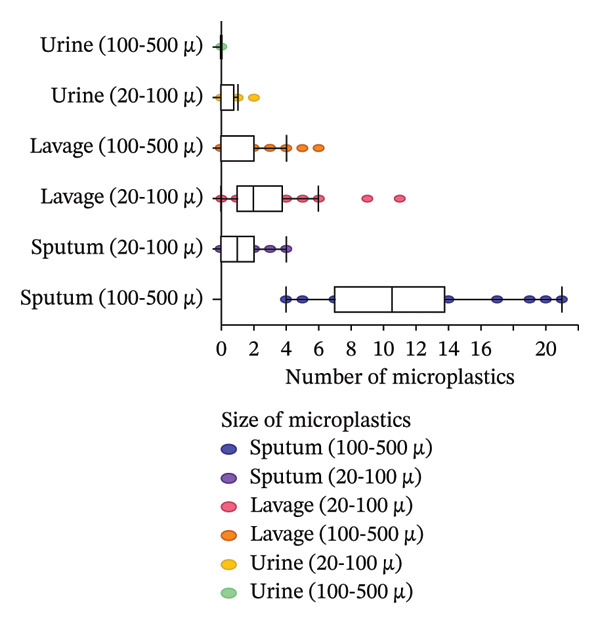
MP size in human urine, sputum, and lavage samples.

**FIGURE 3:**
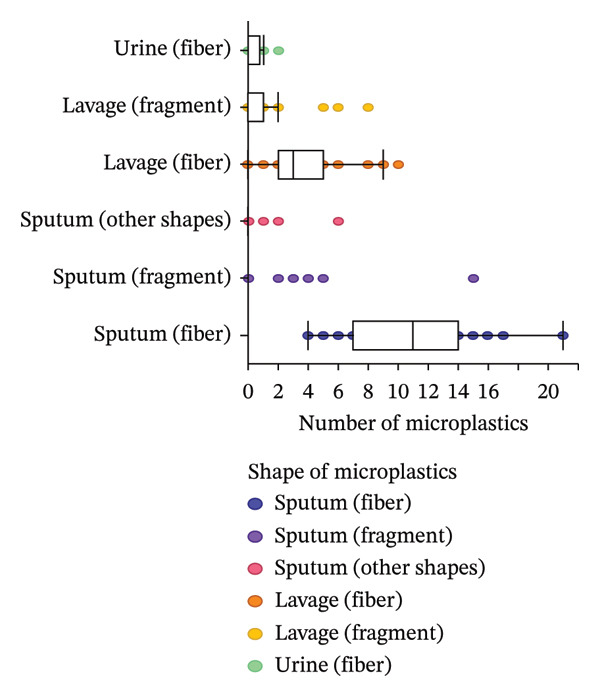
MP shape in human urine, sputum, and lavage samples.

In addition to the differences in total particle counts, the MPs in each biological matrix also differed in size, shape, and polymer type. MPs in urine were almost exclusively < 50 µm, consistent with the hypothesis of renal selective excretion of smaller particles. Particles in sputum and BALF samples appeared to be of a greater size range, with some particles > 100 µm. This finding is likely explained by MP deposition within the upper airways and alveolar spaces within the respiratory tract and the particles being naturally removed through mucociliary clearance mechanisms (Figure 2). Morphologically, fibrous forms were the dominant shape in sputum‐ and BALF‐based sampling. This may be explained by the inhalation of airborne synthetic fibers which are often shed from clothing and household dust—that stay suspended in air and are inhaled deeper within the lungs. In contrast, urine samples had, on average, more fragments than fibers. This suggests that MPs ingested through food routes, which are mediated by gastrointestinal absorption and systemic distribution to other sites, are preferentially excreted through renal pathways as nonfibrous forms (Figure 3).

Polymer analysis also demonstrated significant differences. The BALF exhibited the greatest diversity of polymer types including PE, polypropylene, PS, polyethylene terephthalate, and polyvinyl chloride. The broad spectrum demonstrates a greater depth of lung exposure of an environmental mixture of heterogeneous MPs. Urine samples had the least variety of polymers, which corroborates the reality of renal excretion limited to a narrow window of small‐sized, bioavailable polymers. These findings emphasize the importance of reporting the presence of MPs and their physicochemical properties when considering the human exposure pathways and their potential health effects.

### 3.3. Operational Considerations

In addition to variation in the sensitivity of detection and the properties of MPs, functional and methodological aspects also affect the suitability and applicability of biological matrices for human biomonitoring. These aspects include simplicity of sample collection, risk of contamination, volume of sample required, and ethical considerations. Urine samples are minimally invasive and relatively easy to obtain in bulk, which is useful for both population studies and sequential sampling in follow‐up studies. However, due to renal filtration barriers and particle size selection during urine formation, urine samples may not adequately represent exposure to inhaled MPs with low systemic circulation potential (e.g., larger and fibrous‐shaped particles). Sputum can be obtained by voluntary deep coughing, which allows for less‐than‐invasive assessment of exposure in the upper airway. Sputum samples are well suited for short‐term monitoring of inhalation exposures to airborne particulates (particularly fibrous MPs); however, quantity (e.g., dry versus wet sputum) and quality (e.g., grade of sputum) will differ substantially between subjects, based on each subject’s respiratory condition, efforts, and mucous output. Changes in sputum quality and quantity will create challenges for quantification and comparison between subjects. Finally, BALF samples the lower respiratory tract and alveolar compartments and therefore represents inhaled deposits. Access to BALF is direct but is obtained using invasive bronchoscopy.

BALF usually shows considerable MP diversity and higher particle translocation; hence, it is very relevant in mechanistic and clinical research contexts. However, the invasive nature limits its practical use to clinical samples with a low sample size that does not lend itself to generalizability. Moreover, BALF sampling needs to be done in controlled sterile environments to prevent environmental contamination. These logistical and ethical compromises must be balanced alongside considerations of analytical performance when choosing a biological matrix for MP exposure assays.

### 3.4. Comparative Scoring Framework

To provide a systematic evaluation of the biomonitoring potential of each biological matrix, we developed a simplified MCDA framework. Although the classic AHP involves pairwise comparisons and consistency ratio validation, our approach adopted a simplified scoring system inspired by AHP principles, without performing full pairwise matrix weighting.

Seven key criteria were identified based on biological relevance, analytical feasibility, and logistical practicality: detection sensitivity, polymer diversity, ease of collection, noninvasiveness, biological representativeness, analytical interference (e.g., matrix contamination or background noise), and repeatability. Each matrix was scored on a scale from 1 (least favorable) to 5 (most favorable) for each criterion.

The scores were assigned based on a combination of empirical data from our dataset and expert judgment from previous literature and laboratory experience. Equal weight was given to each criterion to maintain simplicity and transparency, acknowledging that future studies may adopt weighted systems based on specific research priorities.

**FIGURE 4:**
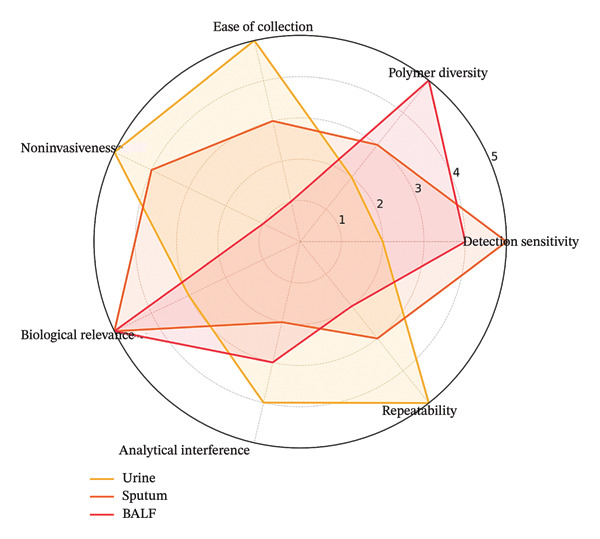
Radar chart comparing the relative performance of urine, sputum, and BALF across seven biomonitoring criteria. Each axis is scaled from 1 (least favorable) to 5 (most favorable). The outer ring (score 5) represents optimal performance. Higher scores indicate better suitability for that criterion. This visualization enables side‐by‐side comparison of matrix‐specific strengths and weaknesses.

**FIGURE 5:**
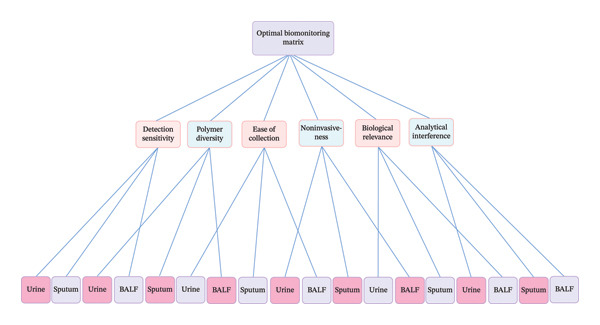
Hierarchical structure of decision‐making framework (AHP‐inspired) for selecting the optimal biological matrix for MP biomonitoring, based on seven scientific and operational criteria.

A visual summary of the multicriteria scores is presented in a radar chart (Figure 4), enabling side‐by‐side comparison of the three matrices across all evaluation domains. Additionally, the hierarchical structure of the decision‐making process is illustrated in Figure 5, outlining the logical flow and weighting rationale behind matrix selection using an AHP‐inspired framework.

### 3.5. Interpretative Summary

MPs were most commonly identified in sputum. However, it was urine that topped the multicriteria decision model. That question largely reflects the greater accessibility, noninvasiveness, and replicability of the sampling process in urine, which are key features when working with large populations or through long periods of time. In contrast, BALF, which has the only contact with the deep areas of the lungs and might have had a greater variety of polymers with more invasive features and logistical concerns, came at the lowest in composite scores.

Such results indicate that the choice of a biological matrix must be influenced by study goals and settings. For instance, urine would be most compatible with population‐wide screening on exposure and longitudinal follow‐up. In contrast, sputum—sensitive extensively in detection—would best fit analyses specifically targeting respiratory exposure to inhaled MPs. BALF, limited by clinical scope and anatomical significance, is best kept for mechanistic, diagnostic, or case‐specific work carried out in a controlled clinical setting.

Generally, this work employs the integrative scoring system that makes it possible to convert unprocessed observational data into structured and usable decision‐making recommendations. Such comparative approaches are instrumental in optimization of study design in future research on human MP exposure.

## 4. Discussion

This study provides a comparative evaluation of the three biological matrices—urine, sputum, and BALF—of human biomonitoring of MPs by detection ability, physical characteristics, polymeric types, and operational practicality. The goal was to provide a systematically assessed database to facilitate the selection of the appropriate matrix for future studies depending on the target under analysis. The results show that median MP concentrations were greatest in sputum samples, consistent with earlier reports showing the respiratory tract—specifically upper airways—being a key site for deposition of inhaled MPs. The high common occurrence of fibrous materials in sputum and BALF supports the early hypothesis that air fibers are a predominant fraction of inhaled MPs [26, 30]. On the other hand, urine MPs were shorter and were more usually presented as fragments not fibers and indicated systemic redistribution and renal elimination of inhaled or ingested material.

Polymer variety assessments further indicated BALF had the widest range of polymer types, possibly due to its potential to reach deeper lung regions and long‐term deposition of particles able to persist. However, based on this analysis, BALF is still less favored despite having a better analytical framework, as collecting BALF is an invasive measure, limiting the ability to do research outside of clinical or community‐based studies. MCDA, and more specifically the AHP, has been extensively applied in environmental health problems, from prioritizing pharmaceutical removal options in hospital wastes, to assessing the most relevant medical waste management options, and ecological hazardous regulation like toxic algal blooms, based on technical, environmental, and social criteria [34, 35]. The variety of decision‐support applications inspired by these uses into this work is an AHP‐like scoring system formatted to compare and rank biomonitoring matrices based on concern for MP exposures with practical consideration blended with analytical performance. Methodologically, our parsimonious scoring scheme following AHP design provided a systematic approach based on seven criteria: detection sensitivity, polymer variety, collecting ease, noninvasiveness, biological relevance, analytical interference, and repeatability.

Even though this framework does not use pairwise weighting or hierarchical optimization, it provides a clear and flexible framework appropriate for rapid appraisal and decision‐making. This process consolidated existing knowledge from the literature and incorporated new interpretive scoring, allowing us to generate a matrix‑specific profile and evaluate its analytical usefulness for practical applications. Urine is also the most scalable and feasible matrix with many longitudinal and population‐based large‐size studies previously completed. The collection of urine is logistically easy and can be repeated, and it is noninvasive, making it appropriate for occupational exposure, epidemiology surveillance programs, and for screening at‐risk populations. However, urine is less diversely polymerized and has relatively low sensitivity of detection that means urine often underestimates overall exposure, particularly exposure to inhaled MPs (Table 2).

**TABLE 2:** Comparative strengths and limitations of urine, sputum, and BALF as biomonitoring matrices for MPs.

Criterion	Urine	Sputum	BALF
Detection sensitivity	Low to moderate; may miss larger or retained particles	High; effectively captures inhaled fibrous MPs	Moderate to high; deep lung sampling enables broader capture
Polymer diversity	Limited; fewer polymer types detected	Moderate; better representation of inhaled MPs	High; includes wide polymer spectrum, often retained longer
Particle size range	Mostly < 50 µm; renal filtration limits detection of larger MPs	Broad range; up to > 100 µm common	Broadest range; includes very fine and larger particles
Matrix interference	Generally low	Moderate; presence of mucus may interfere	High; proteinaceous fluid may require pretreatment
Repeatability/stability	High; consistent across populations	Moderate; quality depends on patient effort	Low; invasive and not repeatable outside clinical settings
Ease of collection	Very easy; noninvasive and widely acceptable	Moderate; requires spontaneous or induced sputum	Difficult; requires bronchoscopy under clinical conditions
Ethical/logistical burden	Minimal; suitable for large‐scale studies	Acceptable; field‐feasible	High; ethical clearance and hospital infrastructure needed
Ideal use case	Population surveys, chronic exposure, screening	Respiratory studies, occupational exposure assessments	Clinical research, mechanistic studies
Composite score	26	25	21
Study type recommendation	Epidemiological/screening	Occupational/urban exposure	Mechanistic/clinical research

*Note:* Analytical and practical dimensions are based on data from Jahedi et al. [33] and supported by Huang et al. [36], Qiu et al. [31], and Rotchell et al. [27]. Composite scores (out of 35) were derived from the multicriteria decision framework described in Section 2.4.

Sputum provides a balanced option, combining relatively high detection sensitivity with moderate feasibility. It captures MPs deposited in the upper respiratory tract, particularly fibrous types associated with inhaled airborne particles. Although it requires active patient cooperation (e.g., spontaneous or induced coughing), it remains noninvasive and does not demand clinical infrastructure. This makes sputum an ideal choice for midscale respiratory exposure assessments or studies focused on urban or occupational air pollution environments.

BALF offers unmatched anatomical specificity and deep‐lung insight, often detecting a broader polymer spectrum and larger particle sizes not seen in other matrices. This makes it particularly valuable in mechanistic or diagnostic studies where spatial resolution and polymer characterization are critical—such as in research on chronic respiratory diseases or inhalation toxicology. Nevertheless, its invasiveness, cost, and ethical constraints limit its use to clinical settings or controlled research cohorts.

Therefore, the choice of matrix should be purpose‐driven: urine for ease and scalability, sputum for respiratory‐focused screening, and BALF for in‐depth clinical investigations. A tiered or multimatrix approach may also be valuable in capturing both systemic and compartmental exposure profiles in comprehensive studies.

The major limitation of this study is the reliance on secondary data without reanalysis of raw MP quantification datasets. Also, variations in analytical protocols, contamination controls, and detection thresholds across the original studies may introduce bias. Future research should prioritize harmonized protocols and consider cross‐matrix sampling within the same cohort to validate these comparative trends.

In conclusion, the choice of biological matrix should be guided by the specific goals, resources, and ethical constraints of each study. This work contributes a decision‐oriented comparative framework that can support the design of robust and targeted human MP exposure assessments.

## 5. Conclusion

This study provides a structured, empirical framework for comparative assessment of biological matrices for human MP biomonitoring. As demonstrated, no single matrix is universally superior; rather, matrix selection depends on study objectives, resource availability, and ethical considerations. Urine is most suitable for large‐scale population studies and noninvasive longitudinal monitoring. Sputum is preferred for targeted respiratory exposure assessments, particularly for airborne fibers. BALF is optimal for detailed clinical or mechanistic investigations requiring deep‐lung anatomical specificity.

The application of our scoring system and hierarchical decision analysis offers a reproducible and transparent method for matrix selection in future biomonitoring studies. As MP pollution is an emerging public health concern, these adaptive, purpose‐driven strategies can support reliable data generation, evidence‐based regulation, and ultimately, human health protection. Future research should prioritize protocol harmonization across laboratories and consider integrated multimatrix approaches to fully characterize exposure profiles across biological compartments. Validation of our findings in larger, healthy cohorts is also recommended.

### 5.1. Limitations

The sample size (*n* = 30) is relatively modest. However, this cohort is unique in providing matched urine, sputum, and BALF samples from the same individuals. The invasive nature of bronchoscopy limits larger sample collection in healthy populations. Nevertheless, our findings should be validated in larger, multicenter studies that include healthy controls. The nonparametric statistical tests used are appropriate for this sample size.

## Author Contributions

Faezeh Jahedi: writing–review and editing, supervision, project administration, methodology, investigation, formal analysis, and conceptualization. Neamatollah Jaafarzadeh Haghighi Fard: writing–review and editing, writing–original draft, supervision, investigation, funding acquisition, and conceptualization. Mehdi Ahmadi: writing–review and editing and supervision. Afshin Takdastan: supervision and investigation.

## Funding

This research was financially supported by both the Iran National Science Foundation (INSF, grant no. 4031900) and Ahvaz Jundishapur University of Medical Sciences (project code: ETRC‐0302).

## Disclosure

This paper is part of the PhD dissertation of Faezeh Jahedi (project code: ETRC‐0302) at Ahvaz Jundishapur University of Medical Sciences.

## Ethics Statement

The authors have nothing to report.

## Consent

The authors have nothing to report.

## Conflicts of Interest

The authors declare no conflicts of interest.

## Data Availability

The data that support the findings of this study are available on request from the corresponding author. The data are not publicly available due to privacy or ethical restrictions.
